# Neutrophil Extracellular Traps Promote NLRP3 Inflammasome Activation and Glomerular Endothelial Dysfunction in Diabetic Kidney Disease

**DOI:** 10.3390/nu14142965

**Published:** 2022-07-20

**Authors:** Anubhuti Gupta, Kunal Singh, Sameen Fatima, Saira Ambreen, Silke Zimmermann, Ruaa Younis, Shruthi Krishnan, Rajiv Rana, Ihsan Gadi, Constantin Schwab, Ronald Biemann, Khurrum Shahzad, Vibha Rani, Shakir Ali, Peter Rene Mertens, Shrey Kohli, Berend Isermann

**Affiliations:** 1 Institute of Laboratory Medicine, Clinical Chemistry and Molecular Diagnostics, Universitätsklinikum Leipzig, Leipzig University, 04103 Leipzig, Germany; anubhuti.gupta@medizin.uni-leipzig.de (A.G.); kunal.singh@medizin.uni-leipzig.de (K.S.); sameen.fatima@medizin.uni-leipzig.de (S.F.); saira.ambreen@medizin.uni-leipzig.de (S.A.); silke.zimmermann@medizin.uni-leipzig.de (S.Z.); ruaa.12.7@gmail.com (R.Y.); shruthi.krishnan@med.ovgu.de (S.K.); rajiv.rana@medizin.uni-leipzig.de (R.R.); ihsan.gadi@medizin.uni-leipzig.de (I.G.); ronald.biemann@medizin.uni-leipzig.de (R.B.); khurrum.shahzad@medizin.uni-leipzig.de (K.S.); shrey.kohli@medizin.uni-leipzig.de (S.K.); 2 Institute of Pathology, University of Heidelberg, 69120 Heidelberg, Germany; constantin.schwab@med.uni-heidelberg.de; 3 Department of Biotechnology, Jaypee Institute of Information Technology, Noida 201309, Uttar Pradesh, India; vibha.rani@jiit.ac.in; 4 Department of Biochemistry, School of Chemical and Life Sciences, Jamia Hamdard University, New Delhi 110062, India; sali@jamiahamdard.ac.in; 5 Clinic of Nephrology and Hypertension, Diabetes and Endocrinology, Otto-von-Guericke University, 39120 Magdeburg, Germany; peter.mertens@med.ovgu.de

**Keywords:** diabetic kidney disease, neutrophil extracellular traps, NLRP3 inflammasome, endothelial dysfunction, glomerular filtration barrier disruption, glomerular endothelial cells

## Abstract

Diabetes mellitus is a metabolic disease largely due to lifestyle and nutritional imbalance, resulting in insulin resistance, hyperglycemia and vascular complications. Diabetic kidney disease (DKD) is a major cause of end-stage renal failure contributing to morbidity and mortality worldwide. Therapeutic options to prevent or reverse DKD progression are limited. Endothelial and glomerular filtration barrier (GFB) dysfunction and sterile inflammation are associated with DKD. Neutrophil extracellular traps (NETs), originally identified as an innate immune mechanism to combat infection, have been implicated in sterile inflammatory responses in non-communicable diseases. However, the contribution of NETs in DKD remains unknown. Here, we show that biomarkers of NETs are increased in diabetic mice and diabetic patients and that these changes correlate with DKD severity. Mechanistically, NETs promote NLRP3 inflammasome activation and glomerular endothelial dysfunction under high glucose stress in vitro and in vivo. Inhibition of NETs (PAD4 inhibitor) ameliorate endothelial dysfunction and renal injury in DKD. Taken together, NET-induced sterile inflammation promotes diabetes-associated endothelial dysfunction, identifying a new pathomechanism contributing to DKD. Inhibition of NETs may be a promising therapeutic strategy in DKD.

## 1. Introduction

Diabetes mellitus is a lifestyle- and malnutrition-based metabolic disease that has reached the proportion of a worldwide pandemic. Diabetes mellitus is primarily hallmarked by insulin deficiency (type 1 diabetes mellitus) or insulin resistance (type 2 diabetes mellitus—the predominant diabetes form) and hyperglycemia in association with micro-and macro-vasculature dysfunction [1,2]. While interventions to improve diet and physical activity are important therapeutic pillars in controlling type 2 diabetes mellitus [3], their success is limited, and the increasing prevalence of secondary vascular complications are a major cause of morbidity and mortality in affected patients. 

Diabetic kidney disease (DKD) is a major microvascular complication of diabetes mellitus [4]. Despite the recent progress, effective therapies to prevent, halt or even reverse DKD are lacking. Albuminuria, an earlier marker of DKD in most affected patients, indicates not only an increased risk for renal failure, but also cardiovascular complications [5]. A common underlying pathomechanism is thought to be endothelial dysfunction. DKD and cardiovascular complications are also both closely linked with sterile inflammation [6,7]. The extent to which endothelial dysfunction and sterile inflammation are mechanistically linked remains largely unknown. One inflammatory feature in DKD is increased neutrophil activation [8], yet the mechanism through which activated neutrophils orchestrate vascular injury in DKD remains to be shown. 

Neutrophil activation results in the decondensation of DNA and histone citrullination by histone deaminases such as peptidyl arginine deiminase 4 (PAD4) [9]. This leads to the secretion of neutrophil extracellular traps (NETs), comprising DNA, histones and neutrophil proteases such as neutrophil elastase (NE) and myeloperoxidase (MPO) [10,11,12,13]. While NETs were originally linked to the control of infectious pathogens [14,15], more recent studies linked NET formation and deposition with sterile inflammation in non-communicable diseases such as cardiovascular diseases and acute kidney injury, e.g., glomerulonephritis [16,17,18]. In the setting of acute kidney injury, NETs impair vascular integrity and promote renal damage [19,20]. As NETs may cause endothelial cell injury, their formation may promote endothelial dysfunction in non-communicable diseases [21,22,23,24]. 

NETs interact with the NRLP3 inflammasome, another mechanism of sterile inflammation, in cardiovascular disease such as atherosclerosis and deep vein thrombosis (DVT) [25,26,27]. NLRP3 inflammasome activation has been mechanistically lined to DKD [28]. However, (i) whether NETs have a pathogenic function in DKD, (ii) whether NLRP3 inflammasome and NET formation interact in the pathogenesis of DKD, and (iii) whether NETs contribute to glomerular endothelial dysfunction in DKD all remain unknown. These open questions were addressed in the current study using a combination of in vitro and in vivo approaches. 

## 2. Methods 

### 2.1. Mice 

Wild type (C57BL/6J) were bred at the local animal facility or were obtained from Janvier (Le Genest-Saint-Isle, France). Age-matched, 8-week-old littermates were randomly assigned to intervention or control groups. Hyperglycemia was induced by administering low dose of streptozotocin (STZ, 60 mg/kg, dissolved in 0.05 M sterile sodium citrate, pH 4.5) on 5 consecutive days intraperitoneally (i.p.) in mice [29,30]. Mice were considered diabetic if blood glucose levels were above 300 mg/dL. Persistent hyperglycemia was maintained by checking blood glucose once weekly [30]. A sub-group of mice were injected i.p. either with GSK484 [31] (Cayman, 4 mg/kg body weight, dissolved in DMSO, and diluted in 0.9% NaCl final concentration of DMSO 0.1%) or vehicle. All animal experiments were conducted following standards and procedures approved by the local Animal Care and Use Committee (Landesverwaltungsamt Halle, Germany, no: IKCP-G-04-1542-18 and Institute Animal Ethics Committee as per CPCSEA guidelines, Jamia Hamdard, New Delhi, India, no: JH/CAHF/PL-011). 

Body weight and kidney weight was determined at the time of sacrifice. Blood samples were obtained from the inferior vena cava and collected into syringes prefilled with sodium citrate (final concentration 0.38%). Plasma was obtained by centrifugation at 100× *g* for 10 min followed by 2000× *g* for 10 min. Animals were perfused with ice-cold 0.9% NaCl followed by 4% buffered PFA. The kidneys were collected in methanol stabilized formalin and were fixed for 2 days at 4 °C, embedded in paraffin blocks, and processed for sectioning.

### 2.2. Determination of Albuminuria

Urine from individual mice was collected using metabolic cages. Urinary albumin was determined by ELISA method. Briefly, primary monoclonal antibody of mouse albumin (A90-134A, Goat anti-mouse albumin, Bethyl Laboratories, Montgomery, TX, USA; dilution 1:110) was coated on 96-well plates overnight. Urine was diluted in bovine serum albumin (BSA) and added to respective percolated wells along with standards and blank. This was followed by an incubation for 30 min to allow for binding of albumin to precoated antibodies. Excess unbound albumin was washed, and secondary horse raddish peroxidase (HRP) conjugated antibody specific to core of albumin was added. This albumin–antibodies complex was detected by a 3,3′,5,5′-Tetramethylbenzidine (TMB)-based chromogenic substrate, which was read at 450 nm. Urine creatinine was determined using a commercially available assay of a modified version of the Jaffe method (KGE005, Creatinine Parameter Assay Kit, R&D Systems, Minneapolis, MN, USA). Urinary albumin creatinine ratio (UACR) was calculated by taking the ratio of albumin to creatinine (μg/mg) for each sample. 

### 2.3. ELISA

ELISA for NET markers (NE, H3Cit, and dsDNA) was performed according to manufacturer’s protocol. H3Cit was measured using Cayman’s Citrullinated H3 kit-501620. Mouse neutrophil elastase (NE) was measured using a commercial ELISA from R&D systems (DY4517-05). Quant-iT™ PicoGreen™ dsDNA Assay-Kit (Invitrogen, P7589) was used to measure dsDNA. H3Cit, NE and dsDNA concentrations were determined from their standard curves.

### 2.4. Human Renal Biopsies

Human renal biopsy samples were provided by the tissue bank of the National Center for Tumor Diseases (NCT, Heidelberg, Germany) after obtaining ethical approval (ethic vote no: S-284/2018, Ruprecht-Karls-University of Heidelberg) and informed consent. See Table S1 for patient characteristics.

### 2.5. Immunostaining

Sections were fixed in ice-cold acetone for 1 min after antigen retrieval in Tris-based antigen-unmasking solution. Blocking was performed at room temperature (RT) for 1 h by incubating in phosphate buffered saline (PBS, containing 3% donkey serum and 0.025% Tween 20). The slides were then incubated with primary antibodies H3Cit (1:200, Abcam ab5103), Ly6G (1:200, Abcam ab25377), MPO (1:200, R&D AF3667) or nephrin (1:250, R&D AF3159) or for 48 h at 4 °C. Slides were washed with PBS (3 times) to remove unbound antibody. The sections were then incubated with corresponding fluorescently labeled secondary antibodies (anti-Rb AF546 A10040, anti-goat AF488 A32814 or anti-Rb AF488 A18740) for 2 h followed by three washes in PBS. The kidney sections were mounted using VECTASHIELD mounting medium containing the nuclear stain DAPI (Vectashield plus antifade DAPI, Vector lab H-2000). Images were acquired using a Keyence microscope. The exposure settings and laser gain were kept constant for each condition. A total of 30 fields were captured for each condition, with a single focal plane per field. The analysis was performed using NIH Image-J [32].

### 2.6. PAS Staining

Periodic acid Schiff’s (PAS) staining was carried out based on previously published PAS staining protocol [32,33,34]. First, 5 μm cut paraffin-embedded kidney sections were deparaffinized with xylene and rehydrated with gradients of 95%, 80%, 70%, 50% and 30% ethanol wash. Sections were then oxidized with 0.5% periodic acid solution (10 min) followed by staining by Schiff’s reagent for 20 min. Polysaccharides on sections turned pink after washing under lukewarm water. Sections were then counterstained with hematoxylin. Imaging was performed using Keyence microscope. Fraction mesangial area were calculated using Image-J software by determining pink-colored intensity per glomerulus (40–50 glomeruli per sections), which is the measure of mesangial expansion.

### 2.7. Glomerular Fraction Isolation

C57BL/6 mice with/without intervention were used for glomerular isolation. Both kidneys from the mouse were harvested, and the medulla was removed carefully. The renal cortexes were minced into tiny particles (∼1 mm^3^) in 0.5 mL of Hanks’ Balanced Salt Solution (HBSS, catalog no. 14185052, GIBCO) and digested with collagenase type V (dissolved in HBSS, catalog no. C9263, Sigma, St. Louis, MO, USA) in a water bath at 37 °C for 20 min with pipetting at 5 min intervals. Then, 4 mL of Dulbecco’s Modified Eagle’s Medium (DMEM) supplemented with 10% fetal bovine serum (FBS, catalog no. 10500064, GIBCO) was used to stop the digestion. The digested tissue was spun down at 300× *g* for 1 min at 4 °C and resuspended in 5 mL of Hanks′ Balanced Salt Solution (HBSS). The resulted mixture was transferred onto a prewetted 150 mesh (150 μm) stainless steel cell strainer (catalog no. F513441, Sangon, Shanghai, China). Collected filtrate was transferred in cold HBSS to a prewetted 200 mesh (75 μm) stainless steel cell strainer (catalog no. F513442, Sangon). The resultant filtrate was again passed through with HBSS onto another prewetted 40 μm plastic cell strainer (catalog no. 15-1040, Biologix, Lenexa, KS, USA). The glomerular and tubular fragments retained on the top of the 40 μm strainer were rinsed into a cell culture dish (10 cm in diameter, catalog no. 430167, Corning, Corning, NY, USA). Large fragmented tubules adhere to the bottom of the dish, after 2 min, while leaving the majority of glomeruli and small fragmented tubules floating in the supernatant. This glomeruli-enriched supernatant was collected and passed onto a new prewetted 40 μm strainer to remove the small fragments of tubules. The retained glomeruli on top of the 40 μm strainer was allowed for second adhesion onto a new cell culture dish to further remove the residual large fragments of tubules. The resultant highly purified glomeruli supernatant was collected and centrifuged at 300× *g* at 4 °C and lysed using radio-immunoprecipitation assay (RIPA) buffer to obtain total protein [35].

### 2.8. Cell Culture

Conditionally immortalized mouse and human podocytes and human glomerular endothelial cells (hGENCs) were grown on 10 cm^2^ cell culture plates at 33 °C in the presence of interferon γ (10 U/mL), which enhances expression of the thermosensitive T antigen. These conditions are optimum for cells to proliferate and remain undifferentiated. To induce differentiation, thermoswitching was performed at 37 °C in the absence of interferon γ and the addition of 100 ng/mL vascular endothelial growth factor (VEGF). Experiments were performed after 7 days of differentiation for hGENCs and 14 days of differentiation for mouse and human podocytes. Mouse and human primary glomerular endothelial cells were obtained from cell biologicals, and 2.2 × 10^6^ cells were cultured onto 0.02% gelatin-coated plates at 37 °C in (M-6115 and H-6115 media from cell biologicals). All the cell lines tested negative for mycoplasma. In some experiments, cultured cells were treated with 25 mM glucose (SIGMA, G8769) and/or 10,000 neutrophils, 10 µM GSK484 (Cayman, 17488) for 24 h. Cells cultured under normal glucose (5 mM) or 25 mM mannitol were used as controls.

### 2.9. Neutrophil Isolation

Neutrophils were isolated from human and mice peripheral blood using MACSxpress^®^ Whole Blood Neutrophil Isolation Kit, human (130-104-434) and Neutrophil Isolation Kit, mouse, (130-097-658), Miltenyi Biotec, respectively, following the manufacturers protocol. In co-culture experiments with human neutrophils, a final concentration of 10^6^ cells/mL, and for mouse neutrophils, 2.5 × 10^6^ cells/mL, was used [36].

### 2.10. Immunoblotting

Cell or glomerular lysates were prepared using radioimmunoprecipitation buffer supplemented with protease inhibitor cocktail followed by protein estimation using bicinchonic acid assay (BCA). Approximately 20 μg of protein was loaded per sample and then electrophoretically separated on sodium dodecyl sulfate polyacrylamide gel. Proteins were then transferred to polyvinylidene difluoride membranes (PVDF) and probed with desired primary antibodies IL-β (1:1000, CST 12242S), NLRP3 (1:1000, Novus NBP1-77080SS), pENOS (1:1000, CST 9572), eNOS (1:1000, CST 9571), Nephrin (1:1000, R&D 3159-NN), PAD4 (1:1000, Novus H00023569-M01), H3Cit (1:1000, abcam ab5103), α-tubulin (1:40,000, CST 2144) overnight at 4 °C with gentle shaking. Blots were then washed with TBS supplemented with 0.1% Tween-20 and incubated with horseradish peroxidase-conjugated secondary antibodies. Membranes were developed with the enhanced chemiluminescence system and analyzed using Image-J software.

### 2.11. Barrier Assay

The barrier assay for mimicking the glomerular filtration barrier was carried out in a Boyden chamber system, combined with the fluorescently labeled albumin. Briefly, transwell insert (Greiner, 662641) with 6.5 mm diameter polycarbonate membrane filters of 0.4 µm pore size were used in a 24-well tissue culture plate that forms two compartments. Podocytes (10^4^ cells/well) on the upper compartment and glomerular endothelial cells (3 × 10^4^ cells/well) on the bottom side of the transwell were cultured at 37 °C with 5% CO_2_, forming a confluent monolayer separated by the filter membrane in between. A stable impedance measured over 24 h indicated formation of an intact barrier. The chamber on both sides was treated with 25 mM glucose (control: 25 mM mannitol). In a subset of inserts, supernatant from neutrophils treated with high glucose (25 mM) was exposed on the side of the glomerular endothelial cells for a total of 48 h. After the treatment, the medium was substituted by fluorescently labeled albumin on the upper side of the transwell. After 24 h, the media from the lower compartment were collected, and fluorescently labeled albumin that leaked from the upper compartment was measured by the Cytation 5 image reader. 

Percent barrier integrity was calculated by the formula:

$$\%\;\mathrm{barrier}\;\mathrm{distruption}=\frac{\mathrm{Albumin}\;\mathrm{measured}\;\mathrm{from}\;\mathrm{the}\;\mathrm{bottom}\;\mathrm{of}\;\mathrm{transwell}\;\mathrm{with}\;\mathrm{the}\;\mathrm{cells}\;}{\mathrm{Albumin}\;\mathrm{measured}\;\mathrm{from}\;\mathrm{the}\;\mathrm{bottom}\;\mathrm{of}\;\mathrm{transwell}\;\mathrm{without}\;\mathrm{the}\;\mathrm{cells}}*100\%\;$$

barrier integrity=100−% barrier distruption

### 2.12. Statistical Analysis

Data from individual experiments are expressed as the mean ± standard error of the mean (SEM). To test for differences between groups, Student’s t test or ANOVA was used, with *p* < 0.05 considered as statistically significant. The strength of correlation between groups was tested using the Spearman rank correlation. The Kolmogorov–Smirnov (KS) test or D’Agostino–Pearson Normality test was used to determine whether the data are consistent with a Gaussian distribution. Prism 9 software was used for statistical analyses.

## 3. Results

### 3.1. NET Formation Is Associated with DKD

**Figure 1:**
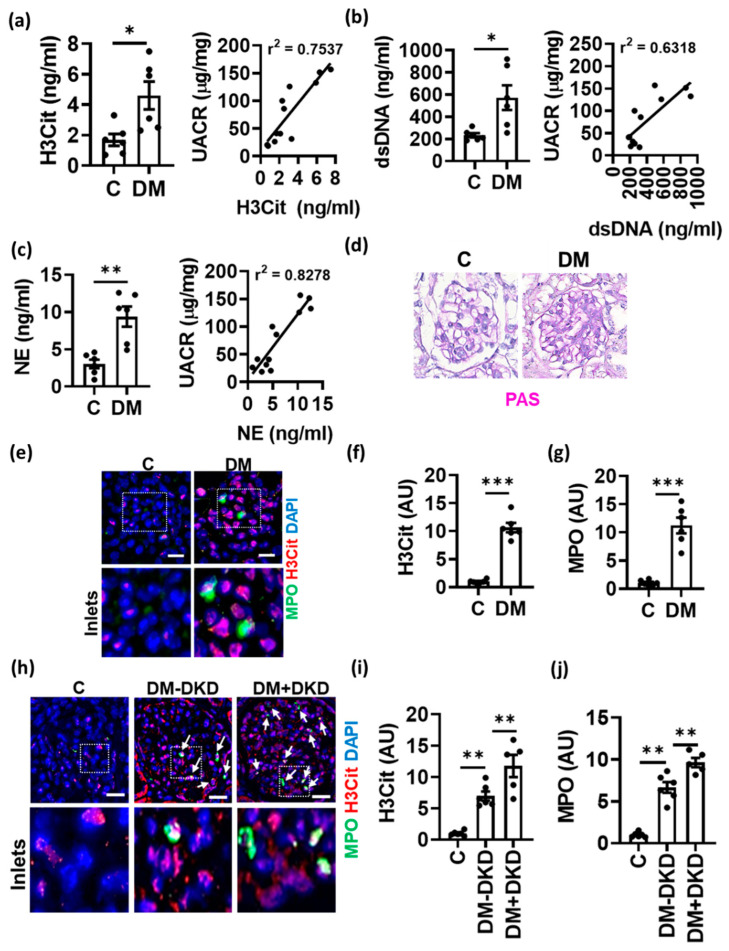
Increased NETs in diabetic kidney disease. (**a**–**c**) Plasma levels of H3Cit, dsDNA and neutrophil elastase, NE (bar graphs with dot-plot) in non-diabetic control (C) and diabetic (DM, STZ-model) mice. (**d**) Representative images showing PAS staining in diabetic (DM) versus non-diabetic control (C) mice. (**e**–**j**) Immunofluorescence staining ((**e**,**h**): representative images; (**f**,**g**,**i**,**j**): bar graphs with dotplot summarizing results) of glomeruli showing H3Cit staining (red) and MPO staining (green) in control (C) and diabetic mice (DM, (**e**–**g**)) and human kidneys from non-diabetic controls (C) and diabetic patients without (DM-DKD) or with (DM+DKD) diabetic kidney disease (**h**–**j**). Correlation (Pearson’s correlation) of H3Cit, dsDNA and NE with urinary albumin creatinine ratio (UACR, line graphs, **a**–**c**). Each dot represents one mouse ((**a**–**c**,**f**,**g**), *n* = 6 mice each group) or one human sample (**i**,**j**); (**e**,**h**): scale bar, 50 μm; (**a**–**c**,**f**,**g**,**i**,**j**): * *p* < 0.05, ** *p* < 0.01, *** *p* < 0.001, *t* test.

To evaluate the role of NETs in DKD, we first determined plasma levels of NET markers using ELISA. Citrullinated histone-3 (H3Cit), neutrophil elastase (NE) and double-stranded DNA (dsDNA) were elevated in both type-1 (STZ, Figure 1a–c) and type-2 (db/db, Figure S1a–c) diabetic mice compared to non-diabetic controls. Furthermore, NET markers positively correlated with albuminuria and fractional mesangial area (Figure 1a–d, Supplementary Figure S1d–f). Immunostaining for NET markers H3Cit and myeloperoxidase (MPO) revealed an increased presence of NETs in the glomeruli of diabetic mice compared to non-diabetic mice (Figure 1e–g). A pathologic relevance of these findings to human diabetic kidney disease is supported by congruent observations made upon immunostaining of H3Cit and MPO in human glomeruli of patients with DKD compared to non-DKD diabetic patients and non-diabetic patients (Figure 1h–j). These results show for the first time an association of NETs with albuminuria and fractional mesangial area (FMA) in DKD.

### 3.2. NETs Induce Inflammasome Activation and Endothelial Dysfunction in Glomerular Endothelial Cells

**Figure 2:**
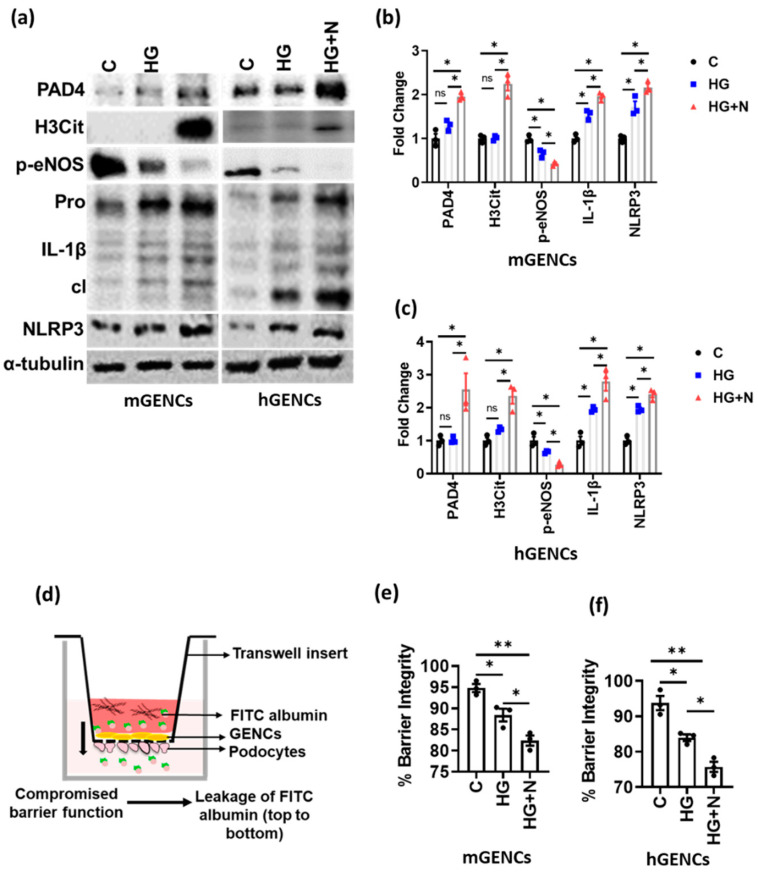
Neutrophils promote inflammasome activation and endothelial dysfunction. (**a**,**b**) Immunoblots ((**a**), representative blots; (**b**), bar graph summarizing results) reflecting NET markers (PAD4, H3Cit), inflammasome activation (IL-1β: cleaved form—cl, inactive preform—Pro; NLRP3) and endothelial function (p-eNOS) in mouse (mGENCs) and (**c**) human (hGENCs) glomerular endothelial cells upon exposure to high glucose and neutrophils (HG+N) compared to high glucose alone (HG, 25 mM) or control (C, PBS). (**d**) Graphical presentation of the Boyden chamber setup for glomerular filtration barrier (GFB) assays where FITC (Fluorescein-5-isothiocyanate) Albumin is used to measure the disruption of GFB. (**e**,**f**) Bar graphs quantifying GFB disruption (reduced barrier integrity) in mGENCs (**e**) and hGENCs (**f**) exposed to high glucose and neutrophils (HG+N) compared to high glucose (HG) alone or control (C, PBS). *N* = 3 independent repeat experiments, each dot represents one independent experiment; * *p* < 0.05, ** *p* < 0.01, ns: non-significant, ANOVA.

As NETs are typically formed in the vasculature in close proximity to endothelial cells, we determined the effect of NETs on glomerular endothelial cell function. Exposure of mouse glomerular endothelial cells (mGENCs) or conditionally immortalized human glomerular endothelial cells (hGENCs) to high glucose (HG) and neutrophils (N) increased the NET markers PAD4 and H3Cit, while high glucose alone had no effect (HG versus HG + N, Figure 2a–c). Additionally, NET formation on GENCs was associated with decreased eNOS phosphorylation (p-eNOS) in vitro, a marker for endothelial dysfunction [37,38], which was more pronounced than the reduction of p-eNOS by high glucose alone, suggesting that NET formation aggravates endothelial dysfunction (Figure 2a–c). 

Diabetes mellitus-associated endothelial dysfunction has been linked to NLRP3 inflammasome activation [37]. While high glucose was sufficient to induce IL-1β and NLRP3 in GENCs, exposure of GENCs to HG and neutrophils (HG+N) resulted in stronger NLRP3 inflammasome activation, as reflected by further increased levels of cleaved IL-1β and NLRP3 (Figure 2a–c). 

To scrutinize the functional consequences of NET formation on glomerular endothelial dysfunction, we assessed the effect of NETs on the function of the glomerular filtration barrier (GFB). GFB was mimicked in vitro by culturing GENCs and podocytes on either side of a transwell insert (Figure 2d). While HG was sufficient for decreasing barrier function as compared to control, exposure to HG and neutrophils (HG+N) impaired barrier function to a higher degree in both human and mouse GFB models (Figure 2e,f). These results suggest that NETs promote glucose-induced GENCs dysfunction. 

### 3.3. PAD4 Inhibition Ameliorates Experimental DKD

**Figure 3:**
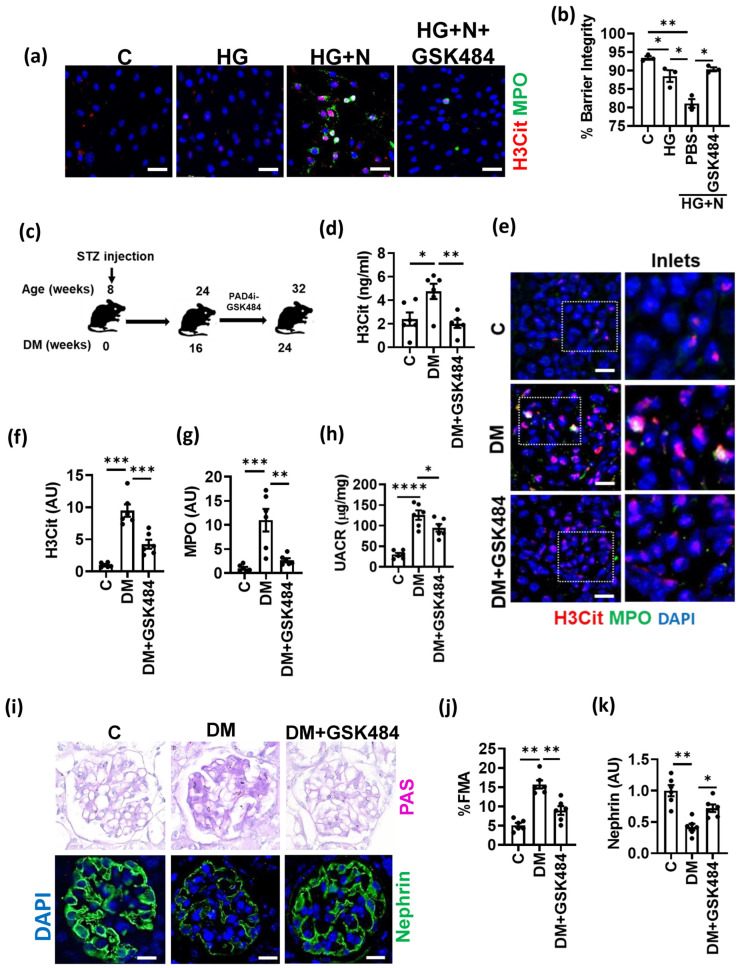
PAD4 inhibition ameliorates NET formation and experimental DKD. (**a**) Representative immunocytochemical images for NET markers MPO (green) and H3Cit (red) on mGENCs exposed to high glucose alone (HG, 25 mM), HG and neutrophils without (HG+N) or with the PAD4 inhibitor GSK484 (HG+N+GSK484) compared to control (C, 5 mM glucose, not neutrophils) mGENCs. (**b**) Bar graph summarizing barrier integrity of the in vitro GFB model. Effect of GSK484-mediated NET inhibition in the presence of high glucose and neutrophils (HG+N+GSK484) compared to control (C), high glucose alone (HG), or HG+N stimulation without GSK484 (PBS). (**c**) Schematic representation of experimental approach of treatment with the PAD4-inhibitor (PAD4i) GSK484 in type-1 diabetic mice (streptozotocin model, STZ) after 16 weeks of established hyperglycemia. (**d**–**g**) Plasma NET markers ((**d**), ELISA, bar graph summarizing results) and NET markers in glomeruli ((**e**), representative immunostaining for H3Cit, red and MPO, green; (**f**,**g**), bar graph summarizing results) in non-diabetic control (C) and diabetic mice without (DM) or with (DM+GSK484) treatment. (**h**–**k**) UACR (**h**), fractional mesangial area ((**i**), top; (**j**), bar graph summarizing results) and nephrin expression ((**i**), bottom, green, DAPI nuclear counterstain, blue; (**k**), bar graph summarizing results) after diabetic mice with (DM+GSK484) or without (DM) GSK484 treatment compared to non-diabetic control mice (C). *n* = 3 independent repeat experiments (**a**,**b**) or *n* = 6 mice each group (**d**–**k**); (**e**,**i**): Scale bar, 50 μm; (**b**,**f**,**g**,**h**,**j**): * *p* < 0.05, ** *p* < 0.01, *** *p*< 0.001, **** *p*< 0.0001, ANOVA.

To address the causality of NET-induced GENCs dysfunction and DKD progression, we used GSK484, a pharmacological inhibitor of PAD4, which inhibits citrullination of histones and thereby NET formation. GSK484 decreased NET formation on mGENCs and hGENCs exposed to HG and neutrophils (Figure 3a and Figure S2a). NET inhibition using GSK484 improved barrier function in cells stimulated with glucose and neutrophils in vitro to levels observed in cells stimulated with glucose alone, establishing that PAD4 inhibition efficiently prevents the NET-dependent effect in vitro (Figure 3b).

To address the in vivo relevance of these findings for DKD, we initiated treatment with GSK484 in diabetic mice after 8 weeks of persistent hyperglycemia and thus after the establishment of albuminuria (Figure 3c). GSK484 treatment was maintained for a further 8 weeks. GSK484 reduced plasma markers of NETs and intraglomerular NET formation (Figure 3d–g, Figure S2b,c), demonstrating that the intervention reduced NET formation in vivo. Reduced NET formation upon GSK484 treatment was associated with reduced albuminuria (Figure 3h) and reduced glomerular injury (Figure 3i–k). Thus, GSK484 reduced the fractional mesangial area while increasing nephrin expression (Figure 3i–k). Taken together, these results show that PAD4 inhibition by GSK484 ameliorates NET formation and improves renal injury in DKD.

### 3.4. PAD4 Inhibition Inhibits NET-Induced Inflammasome Activation and Endothelial Dysfunction

**Figure 4:**
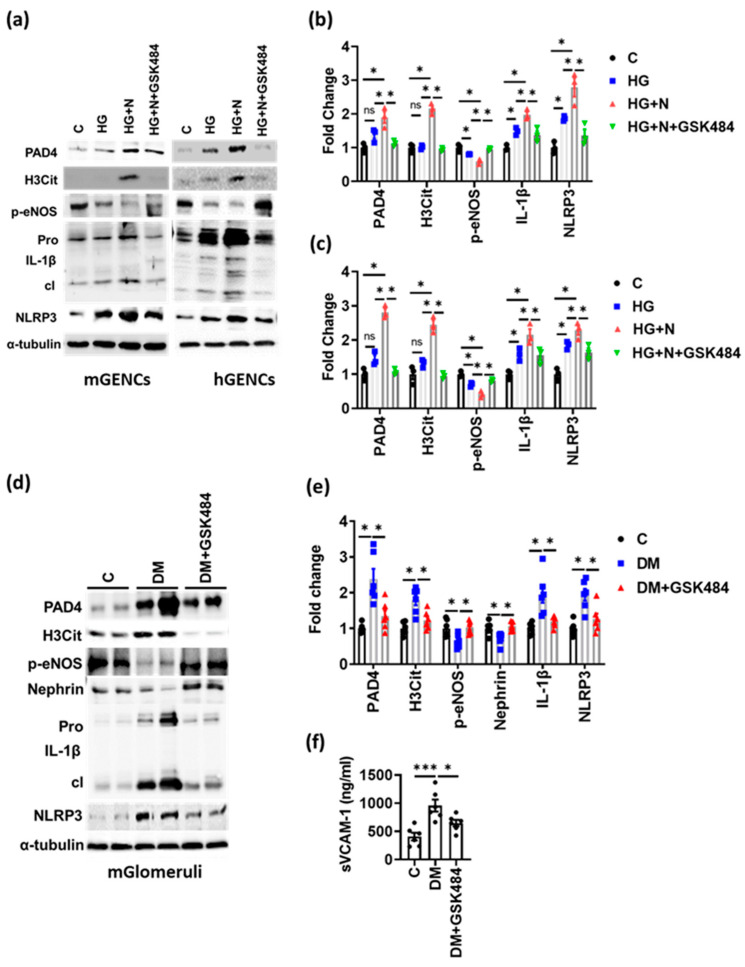
PAD4 inhibition ameliorates inflammasome activation and endothelial dysfunction. (**a**–**c**) Representative immunoblots ((**a**); bar graph summarizing results: (**b**,**c**)) of mGENC (**a**,**b**) and hGENC (**a**,**c**) showing the expression of marker genes for NETs (PAD4, H3Cit), inflammasome activation (IL-1β: cleaved form—cl, inactive preform—Pro; NLRP3) and endothelial dysfunction (p-eNOS) in control cells (C) and cells exposed to high glucose alone (25 mM, HG) or to high glucose plus neutrophils without (HG+N) or with GSK484 (HG+N+GSK484). (**d**,**e**) Representative immunoblots (**d**) and bar graphs (**e**) summarizing results of markers for NETs, inflammasome and endothelial dysfunction (as in (**a**)) in glomerular lysates from diabetic mice without (DM) or with GSK484 (DM+GSK484) compared to non-diabetic controls (C). (**f**) Bar graph summarizing results from ELISA for plasma sVCAM-1 in diabetic mice without (DM) or with GSK484 (DM+GSK484) compared to non-diabetic controls (C). **a**: *n* = 3 independent repeat experiments; (**d**,**f**): *n* = 6 mice in each group; (**a**–**c**,**e**,**f**): * *p* < 0.05, *** *p* < 0.001, ns: non-significant, ANOVA.

We next assessed whether the NET-mediated glomerular injury in DKD depends on inflammasome activation. GSK484 markedly reduced NET markers (PAD4 and H3Cit) and decreased inflammasome activation (expression of cleaved IL-1β and NLRP3), which was associated with improved endothelial function (improved p-eNOS levels) in mGENCs as well as in hGENCs (Figure 4a–c). Similarly, in renal glomerular lysates of diabetic mice receiving GSK484 (Figure 4d,e) markers of NET formation were reduced, which was associated with reduced inflammasome activation (reduced cleaved IL-1β and NLRP3 expression) and increased p-eNOS levels (Figure 4d,e). Furthermore, GSK484 reduced plasma sVCAM-1, suggesting improved endothelial function in vivo (Figure 4f). Thus, inhibition of NET formation in vivo is associated with reduced inflammasome activation and improved endothelial function in renal glomeruli of diabetic mice. 

## 4. Discussion

Neutrophil extracellular traps (NETs) convey pathogenic effects in cardiovascular disease and in acute kidney injury [39,40]. However, their contribution in diabetic kidney disease (DKD) was unknown hitherto. In the current study, we showed that NETs are present within mouse and human diabetic glomeruli and correlate positively with markers of renal damage in DKD. NETs induced by high glucose promote glomerular endothelial dysfunction, which is mechanistically linked with NLRP3 inflammasome activation and IL-1β signaling in glomerular endothelial cells. PAD4 inhibition reduced glucose-induced NETs and prevented endothelial dysfunction, sterile inflammation and renal injury in DKD. These findings highlight a new mechanism of sterile inflammation in DKD, which aggravates renal injury.

NETs, which were originally discovered as a mechanism to control bacterial infection, contribute to sterile inflammation in non-infectious diseases, including acute kidney injury. Increased production or impaired clearance of NETs induce renal injury [41,42,43]. The pathological function of NETs in DKD described here and the associated increase in biomarkers reflecting NET formation are in agreement with previous clinical studies showing increased biomarkers of NETs in diabetic patients with microvascular complications [44,45]. As we observed increased NET formation in glomeruli of diabetic patients, our data support a pathophysiological role in DKD not only in mice, but also in humans. Glomerular NET deposition was not only significantly increased in diabetic patients versus non-diabetic controls but also in diabetic patients with versus diabetic patients with DKD. Future longitudinal studies are needed to determine whether glomerular NET formation precedes clinically detectable DKD and to define the diagnostic value of NET-related biomarkers for the, possible early, detection and stratification of DKD patients. 

The NET-dependent induction or aggravation of reduced eNOS phosphorylation and endothelial barrier integrity paralleled by increased plasma levels of soluble VCAM-1 suggest that NET contributes to endothelial dysfunction in DKD. Endothelial dysfunction, as indicated by albuminuria, reflects systemic endothelial injury and an increased risk for vascular diseases, such as atherosclerosis and myocardial infarction [38,46]. NET formation has been linked with cardio-metabolic diseases such as atherosclerosis, thrombosis, diabetes, obesity and, in this study, DKD [47,48,49]. NET formation is therefore unlikely to be specific for DKD but is more likely a marker of systemic endothelial dysfunction. Thus, we propose that in addition to albuminuria, NET formation is not only a marker for DKD but also for systemic endothelial dysfunction. An assessment of NETs in different tissues is required to address the interaction of neutrophils with other tissue-specific endothelial cell types and other cells that can affect the stability and impact of NETs. Future clinical studies are needed to determine whether NET-related biomarkers are as good as or are better than albuminuria in predicting vascular disease and its complications and whether this holds true only in diabetic patients or also in non-diabetic patients. 

The aforementioned, NET-associated diseases (DKD, atherosclerosis, thrombosis, and obesity) are not only associated with endothelial dysfunction but also with sterile inflammation [28,50,51,52]. In the current study, we identified NET formation as a factor driving NLRP3-inflammasome activation in endothelial cells, thus contributing to sterile inflammation. Inhibition of IL-1β signaling using the IL-1R-antagonist anakinra has been previously shown to reverse kidney dysfunction (albuminuria reflecting podocyte and glomerular filtration barrier dysfunction) [28]. These results suggest a reciprocal interaction of NET formation, sterile inflammation, and endothelial dysfunction, resulting in a vicious cycle. Based on the current results, inhibition of this vicious cycle is possible at several molecular disjunct targets (here, PAD4 or IL-1R inhibition). 

Clinical studies evaluating inflammasome inhibition in patients with chronic inflammatory (rheumatoid arthritis) or vascular (atherosclerosis) diseases revealed an increased risk of infectious diseases [53,54]. Components of NETs, which include citrullinated histones, enzymes (NE, MPO), and dsDNA, are cytotoxic and enhance inflammation. Given the reciprocal interaction of inflammasome and NET formation and based on the current findings, targeting NET formation may constitute an alternative approach to restrict sterile inflammation in DKD and possibly other NET-associated vasculopathies and diseases, without interfering directly with the Nlrp3 inflammasome. Furthermore, new insights into endogenous mechanisms clearing NETs may identify new approaches to restrict NET-associated tissue injury in chronic diseases. 

As pointed out above, NET-induced sterile inflammation impaired glomerular barrier function and reduced nephrin expression, reflecting podocyte dysfunction. These observations suggest that endothelial dysfunction generates a pro-inflammatory micromilieu, which impairs the function in other cell types and promotes organ dysfunction. Crosstalk at the glomerular barrier via, for example, angiopoietins or activated protein C, is established [55,56]. The extent to which the observed podocyte dysfunction is due to IL-1β released from endothelial cells, or secondary due to an increase or lack of other endothelial-derived paracrine signals, remains to be determined in future work. 

Our study has potential limitations. Due to the available animal permissions, we were only able to conduct the intervention studies in mice with streptozotocin-induced hyperglycemia. However, inflammasome activation in DKD has been demonstrated in various diabetes models, including models for type 1 and type 2 diabetes, and in patients with diabetes mellitus [57]. Additionally, NET formation was readily detectable in patients with type 2 diabetes and was higher in type 2 diabetic patients with DKD as compared to those without DKD in the current study. Thus, we expect that our results are also relevant for patients with type 2 diabetes mellitus. Likewise, kinetic studies are needed to determine whether NET formation precedes glomerular damage and thus may trigger the disease process, or rather aggravates the disease once triggered. In either case, based on the current findings, we propose that disrupting the vicious inflammatory cycle of NET formation and inflammasome activation will ameliorate glomerular damage and renal dysfunction. 

## Data Availability

The data that support the findings of this study are available upon reasonable request.

## Supplementary Materials

The following supporting information can be downloaded at: https://www.mdpi.com/2072-6643/14/14/2965/s1, Figure S1: Increased NET formation in db/db mice; Figure S2: Effect of PAD4 inhibition in hGENCs; Table S1: Anthropometric, clinical and metabolic characteristics of renal biopsy control and diabetic patients with and without diabetic kidney disease.

## Abbreviations

DM: diabetes mellitus; DKD: diabetic kidney disease; GFB: dlomerular filtration barrier; NETs: neutrophil extracellular traps; PAD4: protein-arginine deiminase type-4; NE: neutrophil elastase; MPO: myeloperoxidase; IL-1R: interleukin-1 receptor; IL1 β: interleukin 1 Beta; NLRP3: NLR family pyrin domain containing 3; C: control; N: neutrophils; HG: high glucose; H: hour; DVT: deep vein thrombosis; STZ: streptozotocin; i.p.: intraperitoneal; DMSO: dimethyl sulfoxide; NaCl: sodium chloride; HBSS: Hank’s Balanced Salt Solution; FBS: fetal bovine serum; PFA: paraformaldehyde; ELISA: enzyme-linked immunosorbent assay; DMEM: Dulbecco’s Modified Eagle Medium; HRP: horseradish peroxidase; TMB: 3,3′,5,5′-Tetramethylbenzidin; UACR: urine albumin–creatinine ratio; dsDNA: double-stranded DNA; H3Cit: citrullinated histone H3; hGENC: human glomerular endothelial cells; mGENC: mouse glomerular endothelial cells; VEGF: vascular endothelial growth factor; VCAM-1: vascular cell adhesion molecule 1; BCA: bicinchoninic acid; TBS: tris-buffered saline; p-eNOS: phospho-endothelial nitric oxide synthase; FMA: fractional mesangial area.
