# Correction: Gupta et al. Neutrophil Extracellular Traps Promote NLRP3 Inflammasome Activation and Glomerular Endothelial Dysfunction in Diabetic Kidney Disease. *Nutrients* 2022, *14*, 2965

**DOI:** 10.3390/nu15112429

**Published:** 2023-05-23

**Authors:** Anubhuti Gupta, Kunal Singh, Sameen Fatima, Saira Ambreen, Silke Zimmermann, Ruaa Younis, Shruthi Krishnan, Rajiv Rana, Ihsan Gadi, Constantin Schwab, Ronald Biemann, Khurrum Shahzad, Vibha Rani, Shakir Ali, Peter Rene Mertens, Shrey Kohli, Berend Isermann

**Affiliations:** 1 Institute of Laboratory Medicine, Clinical Chemistry and Molecular Diagnostics, Universitätsklinikum Leipzig, Leipzig University, 04103 Leipzig, Germany; 2 Institute of Pathology, University of Heidelberg, 69120 Heidelberg, Germany; 3 Department of Biotechnology, Jaypee Institute of Information Technology, Noida 201309, Uttar Pradesh, India; 4 Department of Biochemistry, School of Chemical and Life Sciences, Jamia Hamdard University, New Delhi 110062, India; 5 Clinic of Nephrology and Hypertension, Diabetes and Endocrinology, Otto-von-Guericke University, 39120 Magdeburg, Germany

**Error in Figure**

In the original publication [1], there was a mistake in **Figure 3e**, as published. The corrected **Figure 3e** appears below. The authors apologize for any inconvenience caused and state that the scientific conclusions are unaffected. This correction was approved by the Academic Editor. The original publication has also been updated.

## Figures and Tables

**Figure 3:**
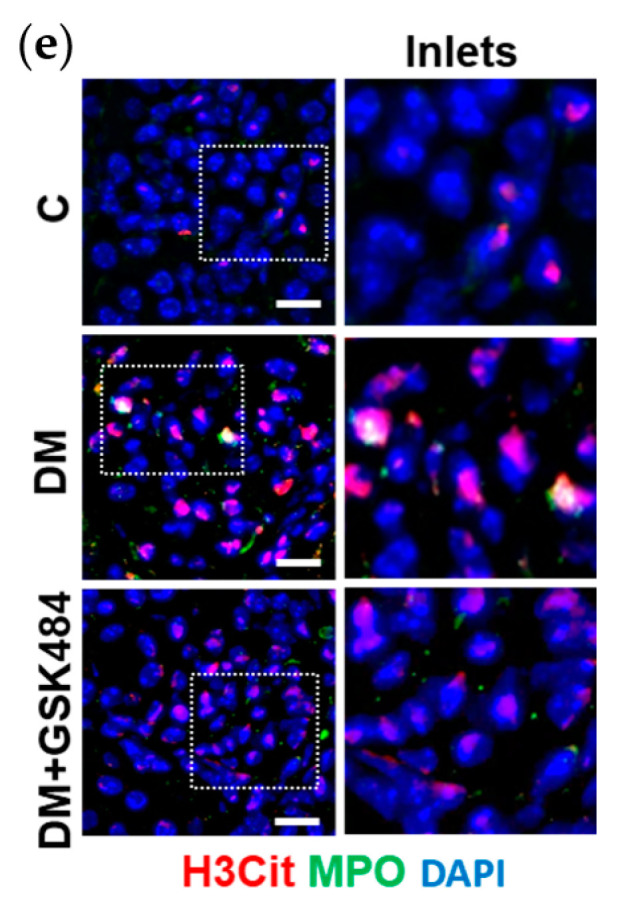
(**e**), representative immunostaining for H3Cit, red and MPO, green.
