# Supplementary material for: Neutrophil Extracellular Traps Promote NLRP3 Inflammasome Activation and Glomerular Endothelial Dysfunction in Diabetic Kidney Disease
Source: Nutrients. 2022 Jul 20;14(14):2965. doi: 10.3390/nu14142965 (PMC9320009; doi:10.3390/nu14142965)
Supplement: Supplementary file 1 [file nutrients-14-02965-s001.zip › nutrients-1813123-supplementary.pdf]

Supplementary Materials

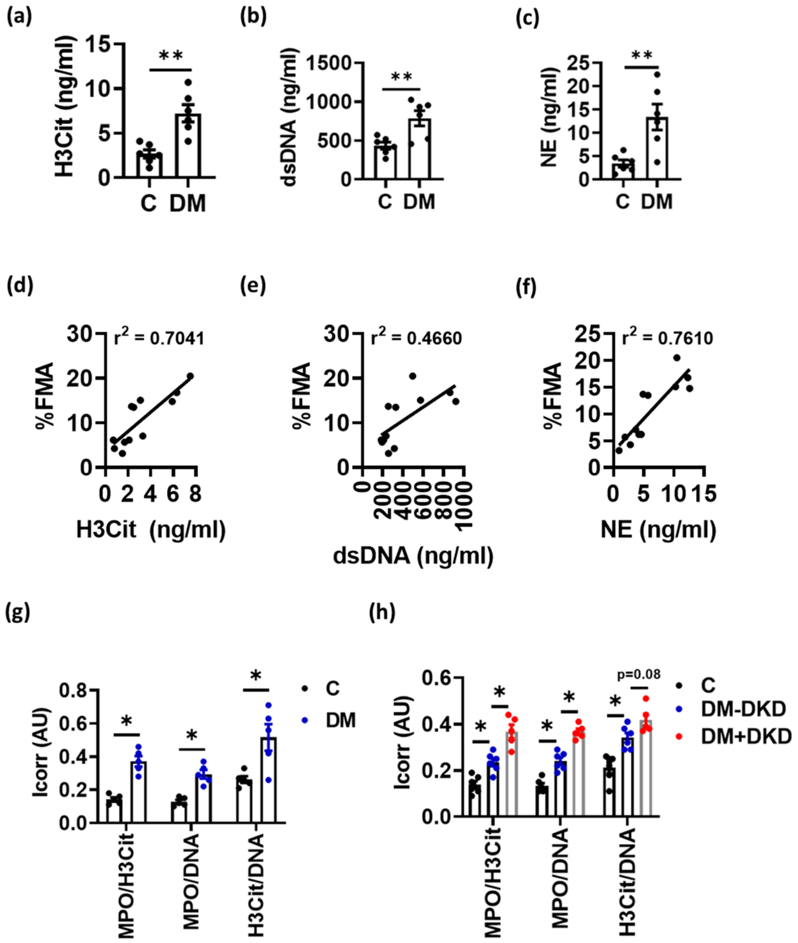

Figure S1. Increased NET formation in db/db mice. (**a–c**) Plasma levels of circulating H3Cit (**a**), dsDNA (**b**) and NE (**c**) measured in non-diabetic (C, db/m mice) and diabetic (DM, db/db model) mice, bar graphs with dot-plot, each dot represent one mouse; ***P*<0.01, t-test. (**d–f**) Correlation (pearson's correlation) of fractional mesangial area (% FMA) obtained from PAS-stained glomeruli of control and DM mice with plasma NET markers H3Cit, dsDNA and NE.(**g–h**) Bar graphs with dot-plot (corresponding to the images in Figure. 1e and 1h.), each dot represent one mouse (**g**) or one patient (**h**) showing index of correlation (Icorr) between different markers (MPO, H3Cit and DNA) showing triple colocalization.

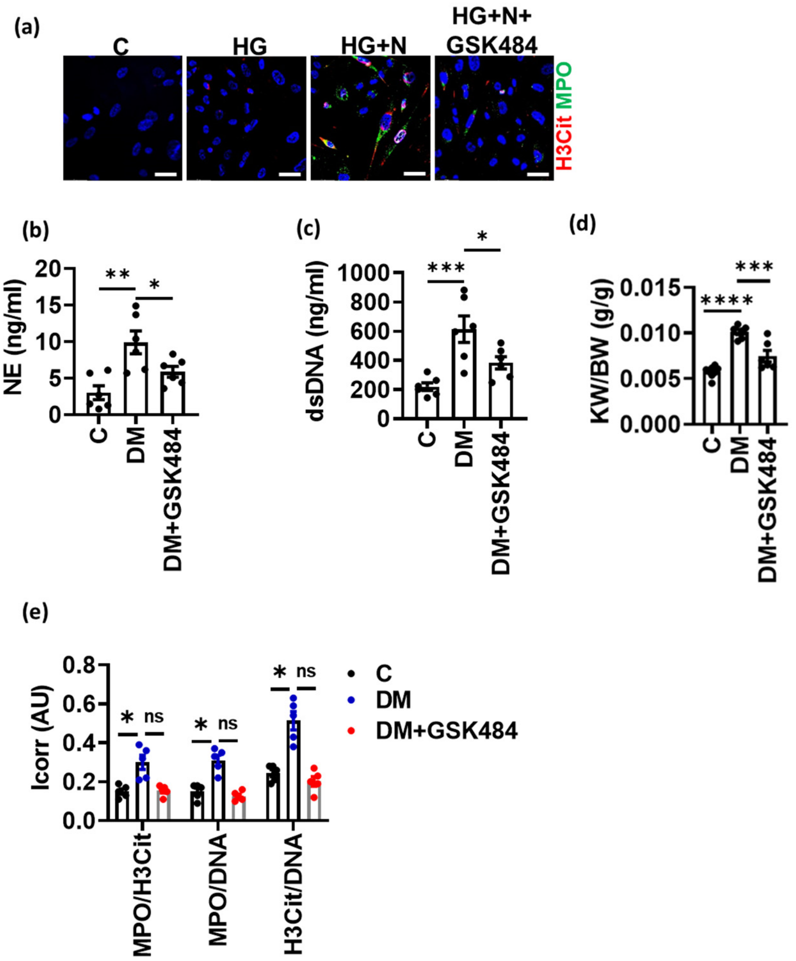

Figure S2. Effect of PAD4 inhibition in hGENCs. (**a**) Representative immunocytochemical images for NET markers MPO (green) and H3Cit (green) on hGENCs exposed to high glucose alone (HG, 25 mM), HG and neutrophils without (HG+N) or with the PAD4 inhibitor GSK484 (HG+N+GSK484) compared to control (C, 5 mM glucose, not neutrophils) hGENCs. (**b**–**d**) Bar graphs summarizing plasma levels of NE (**b**) and dsDNA (**c**) and kidney weight to body weight ratio in non-diabetic control (C) and diabetic mice without (DM) or with (DM+GSK484) treatment. (**e**) Bar graphs with dot-plot (corresponding to the images in Figure. 3e), each dot represent one mouse (**g**) or one patient (**h**) showing index of correlation (Icorr) between different markers (MPO, H3Cit and DNA) showing triple colocalization. *N* = atleast 5 mice each group; **b, c, d**: **p*<0.05, ***p*<0.01, ****p*<0.001, *****p*< 0.0001, ANOVA.

Table S1. (corresponding to Figure. 1h–j). Anthropometric, clinical and metabolic characteristics of renal biopsy control and diabetic patients with and without diabetic kidney disease. Data represents mean ± SEM. Abbreviations: C: Control, DKD: Diabetic Kidney Disease, DM: Diabetes Mellitus, COPD: chronic obstructive pulmonary disease.

**Characteristic**	**C (*n* = 6)**	**DM (-DKD) (*n* = 6)**	**DM (+DKD) (*n* = 5)**
**Age** (years)	71.5 ± 3.1	75.0 ± 3.2	78.2 ± 2.5
**Sex** (female/male)	(3/3)	(3/3)	(2/3)
**History (N) of**			
Hypertension	2	4	4
Brain tumor	1	-	-
COPD	1	-	-
Steatosis hepatitis	1	-	-
peripheral arterial occlusive disease	1	-	-
Hypothyroidism	1	-	-
Type 2 diabetes	-	6	5
**Diabetic complications (N)**			
Diabetic neuropathy	-	2	-
Diabetic kidney disease (DKD)	-	-	5
**Serum creatinine** (mg/dl)	1.1 ± 0.17	1.1 ± 0.06	1.9 ± 0.36
**Serum urea** (mg/dl)	6.6 ± 0.66	6.8 ± 0.69	10.8 ± 1.78
